# A Wide-Bandwidth Inexpensive Current Sensor Based on the Signal Fusion of Tunneling Magnetoresistance and a Current Transformer

**DOI:** 10.3390/s24186071

**Published:** 2024-09-19

**Authors:** Kun Wang, Bin Li, Lixin Wang, Jiafu Wang, Chuansheng Li, Zhiwen Ding, Haiming Shao

**Affiliations:** 1 National Institute of Metrology, Beijing 100029, China; wang-kun@nim.ac.cn (K.W.); wanglix@nim.ac.cn (L.W.); jiafu.wang@nim.ac.cn (J.W.); lichsh@nim.ac.cn (C.L.); dingzhiwen2018@163.com (Z.D.); 2 Prime Science (Beijing) Technology Co., Ltd., Beijing 101102, China; huanmolb@163.com

**Keywords:** current transformer, tunnel magnetoresistance, signal fusion, wide-bandwidth current measurement

## Abstract

In technology and industrial production, many applications require wide-bandwidth current measurements. In this paper, a signal fusion scheme for a current sensor comprising tunneling magnetoresistance and a current transformer is proposed, achieving a flat frequency response in the DC to MHz range. The measurement principles in different cases of the scheme are introduced, and the total transfer function of the entire scheme is derived by analyzing each section separately. Furthermore, the feasibility and selected parameters of the scheme are verified through a systematic simulation utilizing the MATLAB software. Based on the proposed scheme, a group of principal prototypes are built to experimentally evaluate the bandwidth, amplitude and phase flatness, accuracy, sensitivity, and impulse response. The relative amplitude variation in the passband of the fusion sensor is less than 4%, and the estimated bandwidth of the fusion sensor is close to 17 MHz. The accuracy is better than 0.6%, even when measuring the current at 1 MHz, and the relative standard deviation is 5% when measuring the impulse signal. The sensors developed using this scheme, with a low financial cost, have advantages in many wide-bandwidth current measuring scenarios.

## 1. Introduction

Wide-bandwidth current sensors are mainly used to measure periodic high-frequency currents or fast pulse current signals. With the rapid development of technology, high-frequency currents have been broadly applied in the field of manufactory production, grid monitoring, and scientific research. Therefore, accurately measuring and monitoring high-frequency currents has become important and necessary.

For instance, the fast switching currents of recently developed power semiconductors such as GaN require wide-bandwidth current measurements [1,2], with switching current frequencies up to tens of MHz and current consumption at the Ampere level [3]. Moreover, wide-bandwidth current sensors are adopted to measure pulsed current waveforms in lightning monitoring, thereby contributing to assessments of impulse current generators and lightning protection [4]. Wide-bandwidth current sensors are also utilized for signal calibration in the electromagnetic compatibility (EMC) testing of electronic equipment. The basic signals of conducted and radiated EMC are concentrated in the MHz range [5]. In addition, partial discharge detection in cables is indispensable to ensure the safe operation of power grids, where wide-bandwidth current sensors are commonly used to discover discharge currents at frequencies ranging from hundreds of kHz to several MHz [6].

Over the years, conventional current sensors have been developed, including widely applied commercial products such as coaxial current shunts, Hall sensors, and fluxgate sensors. Table 1 presents the characteristics of several current measurement technologies. A common method for measuring current is based on shunt resistors, which have simple structures and can measure high-frequency currents [7,8]. However, shunt resistors do not offer galvanic isolation and can introduce sensing resistance and parasitic inductance, the impacts of which cannot be ignored in applications such as the measurement of semiconductor device switching currents [8].

**Table 1:** Comparison between several current sensors.

Type	Shunt	Current Transformer	Rogowski Coil	Hall Sensor	Fluxgate Sensor	TMR Sensor
Bandwidth	DC~1 MHz	1 kHz~(1~10) MHz	1 kHz~100 MHz	DC~500 kHz	DC~200 kHz	DC~100 kHz
Accuracy	0.01~2%	0.1~2%	0.2~2%	0.5~2%	0.005~0.2%	0.2~1%
Cost	low	low	medium	low	high	low
Galvanic Isolation	no	yes	yes	yes	yes	yes

According to references [9,10,11,12].

Among the current sensors with galvanic isolation, high-frequency current sensors, including current transformers (CTs) and Rogowski coils, have been thoroughly studied [13,14]. These two current sensors behave well in measuring high-frequency currents, even in MHz, but cannot measure lower-frequency signals, especially DC signals [15]. Technologies such as Hall sensors [16], fluxgate sensors [17], and tunneling magnetoresistance (TMR) sensors [18] can also achieve indirect current measurements via magnetic field detection, but their bandwidth is limited, typically below the hundreds of kHz range. Combining these two kinds of sensors allows one to measure current signals from the DC to MHz range [2,19,20]. This combination typically employs principles such as the HOKA method [21] to achieve wideband current signal measurements from DC to high frequency. Although combinations based on the HOKA method achieve broad bandwidth results, their accuracy levels are not sufficient. The HOKA method necessitates additional adders and integrators in the circuit, which increases the complexity of the circuit. In addition, the discrete combination of low- and high-frequency sensors may lead to measurement location inconsistency between the two kinds of sensors.

This study presents an intrinsic signal fusion scheme for wideband current sensors using a low-frequency current sensor (TMR) and a high-frequency current sensor (CT). This scheme is different from the HOKA method and designed with the closed-loop operation of the TMR sensor and the CT. The closed-loop operation given here differs from the simpler existing options for closed-loop magnetoresistance sensors [22,23,24]. The CT in our scheme functions both as a compensation field generator and as a direct current transformer. Previous studies did not consider the effects of current transformation. There is also a lack of investigations focused on the frequency response fusion of the two sensor types considered in this study, namely, the TMR and CT. In our scheme, the total transfer function of the entire signal fusion scheme is derived while simultaneously considering the components of the two sensors. Based on our scheme, we designed a group of fusion sensors with improved structures and additional compensation, achieving a flat frequency response of the fused signal in the DC to MHz range. Additionally, we achieved an accuracy greater than 0.6% in current measurements at 1 MHz and a relative amplitude variation of less than 4% for the passband. Furthermore, measurement of the impulse current signal achieved a 5% relative standard deviation in the impulse amplitude. In addition, the cost of the fusion sensor is limited due to the use of inexpensive components. This proposed scheme works as a comprehensive transfer function analysis reference for the development of a broadband current sensor based on signal fusion, and the scheme has the potential to be broadly applied in wideband current sensors in the fields of technology and industrial production.

## 2. Principles

### 2.1. TMR Low-Frequency Sensor

TMR current sensors outperform other current sensing technologies [23], including shunts, Hall sensors, and fluxgate sensors. TMR sensors were developed based on the Hall sensor with anisotropic and giant magnetoresistance and offer high sensitivity, low power consumption, and high integration [25,26,27].

TMR sensors are based on tunnel magnetoresistance, which takes place in the magnetic tunnel junction [28]. Concerning bandwidth measurements, TMR sensors have the capability to sense current signals from DC to 100 kHz or higher. These characteristics make TMR sensors suitable for use as low-frequency sensors in the proposed signal fusion scheme.

### 2.2. CT High-Frequency Sensor

Current transformers realize current measurements based on the law of electromagnetic induction and Ampère’s circuital law.

**Figure 1:**
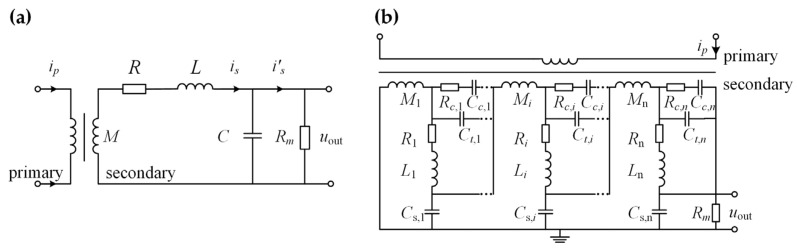
(**a**) The equivalent circuit model of CT. *R*: wire resistance; *L*: leakage inductance; *M*: mutual inductance; *C*: stray capacitance; *R_m_*: load resistor. (**b**) The equivalent circuit model of CT with connected sections, each composed of windings and distributed compensation resistors and capacitors. The subscript *i* means the component of the *i*-th section of the CT; *R_c_*_,*i*_: distributed compensation resistor; *C_c,i_*: distributed compensation capacitor; *C_t,i_*: stray capacitance between different turns of the winding; *C_s,i_*: stray capacitance between the winding and the shield.

Compared with TMR sensors, a CT has the advantage of measuring high-frequency currents in the kHz to MHz range [29]. Thus, a CT is adopted as a high-frequency sensor in the proposed signal fusion scheme. Figure 1a shows an equivalent circuit model of the CT, where *R* represents the wire resistance, *M* represents the mutual inductance, *R_m_* represents the load resistor, *L* represents the leakage inductance, and *C* represents the stray capacitance.

The frequency response function of the CT shown in Figure 1a is acquired using Kirchhoff’s laws and the impedance calculation of *R*, *L*, and *C* in phasor form:

(1) $$\begin{array}{ll} G_{\mathrm{CT}}\left(j\omega \right) & =\frac{U_{\mathrm{out}}\left(j\omega \right)}{I_{p}\left(j\omega \right)}\\ & =\frac{M\cdot j\omega }{-LC\omega^{2}+\left(\frac{L}{R_{m}}+RC\right)\cdot j\omega +\left(\frac{R}{R_{m}}+1\right)} \end{array}$$

Therefore, the corresponding transfer function model of the CT is given as follows:

(2) $$G_{\mathrm{CT}}\left(s\right)=\frac{sM}{LCs^{2}+\left(\frac{L}{R_{m}}+RC\right)s+\left(\frac{R}{R_{m}}+1\right)}$$

Equations (1) and (2) clearly show that the frequency response of the CT can be regarded as a band-pass filter, as explained previously. In comparison, the frequency response of the TMR is like a low-pass filter, so the fused signal of the CT and TMR possibly has a flat response in a broad band from DC to high frequency. In addition, the CTs used in actual applications are equipped with connected sections, each comprising windings and distributed resistors and capacitors for compensation [30,31], to resolve frequency sensitivity issues and signal distortions in high-frequency and pulse applications, as shown in Figure 1b.

### 2.3. Signal Fusion Scheme of the Sensors

**Figure 2:**
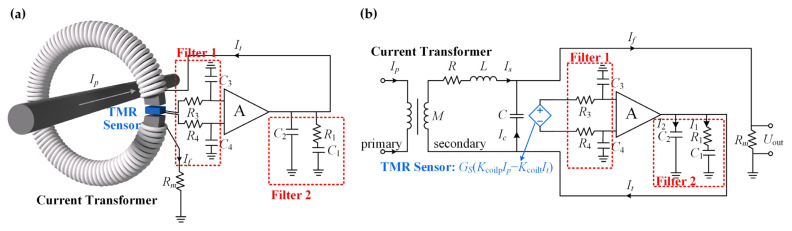
(**a**) Signal fusion schematic for the CT and TMR sensors. *I_p_*: primary current of the CT; A: transconductance amplifier; *I_t_*: the amplified and filtered output current of the TMR sensor; *I_f_*: the output current of the fusion sensor. Here, *I_t_* = *I_f_*. (**b**) The corresponding circuit diagram for the signal fusion scheme. *K*_coilp_: coil constant of the primary coil; *K*_coilt_: coil constant of the secondary coil; *G_s_*: transfer function of the TMR sensor; *I*_2_: current passing through the left branch of Filter 2; *I*_1_: current passing through the right branch of Filter 2; *I_c_*: current passing through the stray capacitance *C* of the CT; *I_s_*: secondary current of the CT; *U*_out_: output voltage.

As shown in Figure 2a, the proposed fusion scheme is composed of a CT and a TMR sensor with its amplification and filtering circuits. In this scheme, the primary current *I_p_* flows through the middle of the CT’s core, which is composed of nanocrystalline material and cut to form a small air gap. A TMR sensor chip is next inserted in the air gap. The output signal of this chip reacting to magnetic field *B_p_* via *I_p_* is filtered, amplified, and filtered again using analog circuits. Then, the processed TMR current signal is sent back as current *I_t_* to the secondary coil surrounding the core and simultaneously produces a voltage signal through the load resistor *R_m_*. In this process, the effect of the CT is divided into two sections. One functions as the feedback loop from the processed TMR current signal to generate the compensation magnetic field *B_t_* in the opposite direction to *B_p_* through the secondary coil, so that the TMR senses the magnetic field difference. The other serves as a current transformer to directly sense *I_p_* and output a voltage signal through *R_m_*. In this way, the signal fusion of the CT and TMR sensor is achieved.

The measurement of the current signal can be considered based on three different cases.

**Case 1**: The primary current *I_p_* is a DC signal or at a comparatively low frequency below the passband of the current transformer. The current transformer does not work, so the output current *I_f_* only comes from the TMR sensor.

**Case 2**: When *I_p_* is at such a high frequency beyond the sensing frequency range of the TMR sensor, the parameters of the capacitors and resistors in the filter are tuned. In this way, the effect of the TMR sensor is filtered out, and the output current *I_f_* simply comes from the current transformer.

**Case 3**: When the frequency of *I_p_* is between the borders in Case 1 and Case 2, the signal of the TMR sensor is amplified, filtered, and superposed on the signal of the current transformer. By adjusting the parameters, the frequency response of the fusion sensor achieves a flat level within the passband.

In Figure 2b, the corresponding circuit diagram is exported from the fusion scheme proposed above and the equivalent circuit model of the CT given in Section 2.2. Especially for convenience, the TMR sensor in the circuit is represented as the current control voltage source *G_s_*(*K*_coilp_*I_p_* − *K*_coilt_*I_t_*) under the control of *I_p_* and feedback *I_t_*. Next, the total transfer function of the entire signal fusion scheme derived from Figure 2b is analyzed section by section, concluding with the complete form.

Firstly, Ampère’s circuital law is applied and the relationship between the magnetic field in the small air gap, *B_p_* and current through the middle of the core, *I_p_*, is acquired as follows:

(3) $$\frac{B_{p}}{\mu_{0}}d+\frac{B_{p}}{\mu_{r}\mu_{0}}\left(l-d\right)=I_{p},$$

where *μ*_0_ is the vacuum permeability, *μ_r_* is the relative permeability of the core, *l* is the average circumference of the core, and *d* is the length of the air gap. Thus, the proportional coefficient is defined as

(4) $$K_{\mathrm{coilp}}=\frac{B_{p}}{I_{p}}=\frac{\mu_{r}\mu_{0}}{\mu_{r}d+l-d},$$

which represents the coil constant of the primary coil. The other coil constant, *K*_coilt_, instead represents the coil constant of the secondary coil, which is used to determine the size of the magnetic field *B_t_* generated by the unit secondary current *I_t_*:

(5) $$K_{\mathrm{coilt}}=\frac{B_{t}}{I_{t}}.$$

The nanocrystalline material adopted for the core has *μ_r_* > 2 × 10^4^, while *d* and *l* are at the cm level. Thus, in Equation (4), the *μ_r_d* >> *l* − *d*. Then, Equation (4) can be simplified as

(6) $$K_{\mathrm{coilp}}\approx \frac{\mu_{0}}{d}.$$

Under ideal conditions, magnetic field *B_t_* fully compensates for magnetic field *B_p_*. Consequently, the following equation is obtained:

(7) $$B_{p}=B_{t},$$

which means that

(8) $$K_{\mathrm{coilp}}I_{p}=K_{\mathrm{coilt}}I_{t},$$

and considering the equilibrium of magnetomotive force,

(9) $$N_{p}I_{p}=N_{t}I_{t},$$

the following equation is derived as

(10) $$\frac{K_{\mathrm{coilp}}}{N_{p}}=\frac{K_{\mathrm{coilt}}}{N_{t}}.$$

In this case, *N_p_* = 1; thus,

(11) $$K_{\mathrm{coilt}}=N_{t}K_{\mathrm{coilp}},$$

and in our scheme, *N_t_* is kept at 200 for the balance of the winding complexity and *B_t_* uniformity.

The transfer function of the TMR sensor can be approximately regarded as a first-order system:

(12) $$G_{s}=\frac{K_{s}}{\tau_{s}s+1},$$

where *K_s_* represents the sensitivity of the TMR sensor measuring the DC current, and *τ_s_* represents the time constant of the TMR sensor.

Next, the transfer functions of Filter 1 and the transconductance amplifier, expressed as *G_f_*_1_ and *G_A_*, respectively, are given using the following equations (supposing *R*_3_ = *R*_4_, *C*_3_ = *C*_4_):

(13) $$G_{f1}\left(s\right)=\frac{1}{sR_{4}C_{4}+1},$$

(14) $$G_{A}\left(s\right)=\frac{K_{a}}{1+\tau_{a}s},$$

where *K_a_* represents the transconductance of the amplifier to the DC voltage signal, and *τ_a_* represents the time constant of the amplifier.

Using Kirchhoff’s current and voltage laws, the equations below are obtained:

(15) $$G_{A}G_{f1}G_{s}\left(K_{\mathrm{coilp}}I_{p}-K_{\mathrm{coilt}}I_{t}\right)=I_{1}+I_{2}+I_{t}$$

(16) $$\frac{I_{2}}{j\omega C_{2}}=I_{1}\left(R_{1}+\frac{1}{j\omega C_{1}}\right)$$

(17) $$\frac{I_{2}}{j\omega C_{2}}=-I_{p}j\omega M+I_{s}R+I_{s}j\omega L+I_{t}R_{m}$$

(18) $$I_{t}=I_{s}+I_{c}$$

(19) $$-I_{p}j\omega M+I_{s}R+I_{s}j\omega L=\frac{I_{c}}{j\omega C}$$

Through mathematical derivation, an equation for the relationship between *I_p_* and *I_t_* can be attained:

(20) $$G_{A}G_{f1}G_{s}K_{\mathrm{coilp}}\left(I_{p}-N_{t}I_{t}\right)=\left(1+\frac{1}{j\omega R_{1}C_{2}+\frac{C_{2}}{C_{1}}}\right)j\omega C_{2}\left(-I_{p}j\omega M+\left(R+j\omega L\right)\times \frac{\frac{I_{t}}{j\omega C}+I_{p}j\omega M}{\frac{1}{j\omega C}+R+j\omega L}+I_{t}R_{m}\right)+I_{t}$$

Next, we solve Equation (20) and consider that the total transfer function of the entire signal fusion scheme is proportional to *I_t_*/*I_p_*:

(21) $$G_{\mathrm{tot}}\left(s\right)=\frac{U_{out}}{I_{p}}=\frac{I_{t}R_{m}}{I_{p}},$$

The total transfer function can then be expressed as

(22) $$G_{\mathrm{tot}}\left(s\right)=\frac{\kappa s^{6}+\lambda s^{5}+\rho s^{4}+\sigma s^{3}+\varphi s^{2}+\chi s+\psi }{\alpha s^{7}+\beta s^{6}+\gamma s^{5}+\delta s^{4}+\varepsilon s^{3}+\zeta s^{2}+\eta s+\theta },$$

which is a seventh-order transfer function. Each coefficient for the power of *s* in the fraction (22) is determined by part or all of the 15 parameters comprising the signal fusion scheme, as presented in Table 2.

**Figure 3:**
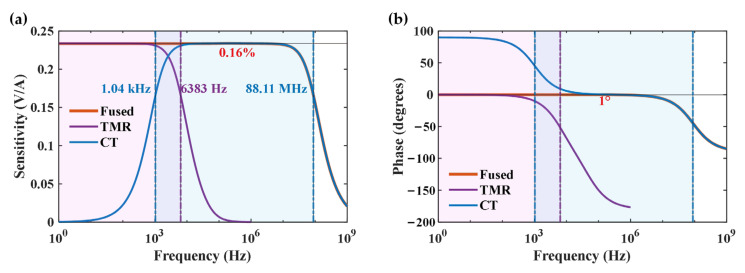
Frequency response simulation results for the proposed signal fusion scheme. (**a**) Simulated amplitude (sensitivity) frequency response. (**b**) Simulated phase frequency response. The meanings of different curves, lines, and regions are the same for both subfigures: The purple curve is the frequency response of the TMR sensor, while the blue curve and the orange curve are the frequency responses of the CT and fused signal, respectively; the purple and blue dash-dotted lines parallel to the *y*-axis represent the bandwidth borders of the TMR sensor and the CT, respectively, which are also annotated using the same color; the regions filled with light purple and light blue represent the passband of the TMR sensor and the CT, respectively. The red annotations in each subfigure represent the variation in the amplitude (phase) frequency response in the passband of the fused signal.

Based on the aforementioned analysis, a simulation was designed to evaluate and verify the proposed transfer function model, utilizing MATLAB R2021b software. In the simulation, each coefficient in Equation (22) is carefully adjusted according to the experimentally acquired value, and the preferred values are given in Table 2. Utilizing the preferred values, a fused signal with a comparatively flat frequency response in the passband is achieved and presented in Figure 3 along with its constituent TMR sensor signal and CT signal.

In Figure 3, the relative variation in the amplitude frequency response (represented by sensitivity) in the passband of the fused signal to the DC amplitude is about 0.16%, and the corresponding variation in the phase frequency response is 1°. In addition, the frequency passband of the fused signal ranges from DC to 88.11 MHz, while the bandwidth of the TMR sensor is about 6.4 kHz. The lower bandwidth of the CT is about 1 kHz, while the upper bandwidth of the CT is the same as that of the fused signal. These simulation results verify the feasibility of the proposed signal fusion scheme.

## 3. Prototype and Experimental Setup

**Figure 4:**
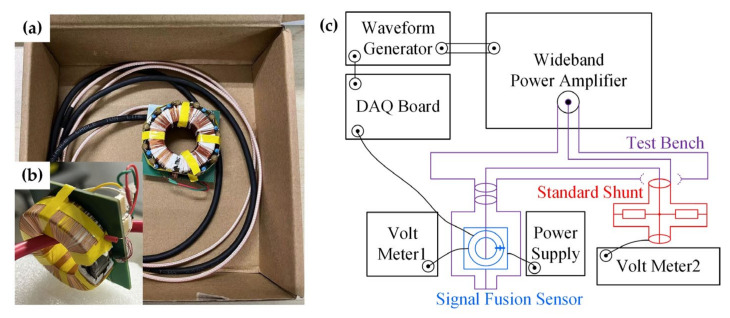
(**a**) The prototype built with sectionally distributed resistors and capacitors for compensation. (**b**) The prototype built without the compensation components. (**c**) Experimental system for evaluating the frequency responses and accuracy of the signal fusion sensor. Each piece of experimental equipment is marked in the picture. DAQ Board: data acquisition board; Volt Meter: voltage meter.

Next, a group of principal prototypes was built for further experimental verification based on the signal fusion scheme described in Section 2, with diagrams and equations and supported by the simulation. Figure 4a presents two different types of prototypes made for comparison, one with sectionally distributed resistors and capacitors (WDRC) and the other without the compensation components (WODRC). The prototypes were built with minimal financial cost due to the inexpensive components selected. The total cost was estimated to be about 25 US dollars for one prototype, as calculated in Table 3.

In addition, the experimental system shown in Figure 4b was established to evaluate the frequency response and accuracy of the signal fusion sensor. A waveform generator is used in this experimental system to offer an input AC or DC signal to the power amplifier, and the power amplifier then generates a magnified current up to 5 A or greater for the test bench. The test bench includes a precision shunt as a measurement standard, with accuracy higher than 10^−6^ class, as well as the signal fusion sensor to be tested.

## 4. Experimental Results and Analyses

**Figure 5:**
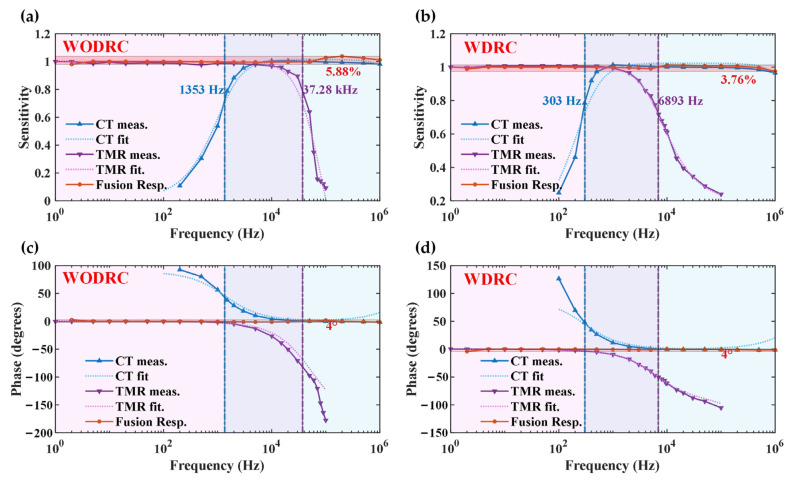
(**a**,**b**): the measured and fit value of the amplitude frequency response (represented by sensitivity) of the CT, TMR sensor, and the fused signal in the WODRC sensor and WDRC sensor, respectively. (**c**,**d**): the measured and fit value of the phase frequency response of the CT, TMR sensor, and the fused signal in the WODRC sensor and WDRC sensor, respectively. The abbreviations below apply to all the subfigures mentioned above. meas.: measured data. fit: fit value using proposed transfer function model. resp.: response (of the fusion sensor). The relative signal variation of the fused signal (orange), and the bandwidths of both the TMR low-frequency sensor (purple) and the CT high-frequency sensor (blue) are given in the annotations.

The frequency responses of the CT, TMR, and fused signal were measured in the experiment and fit using the given model. The results are plotted in Figure 5a,b, present the corresponding amplitude frequency responses (represented by sensitivity) of the WODRC and WDRC sensors, respectively, while Figure 5c,d present the phase frequency response of each sensor.

In the experimental process, the power supply to the TMR sensors of the principal prototypes was first turned off, enabling the frequency responses of the CTs to be evaluated alone. As shown by “CT meas.” and “CT fit”, the fit model was consistent with the measured data in that the coefficient of determination was better than 0.95. Secondly, the signals of the TMR sensors after applying the filter were analyzed separately, with compensation through the secondary coil removed, to assess the sensors’ frequency responses. The results of this section are presented as “TMR meas.” in Figure 5. In addition, the “TMR fit.” presents the fit frequency response, considering the model given in Section 2.3. Next, the signals from the TMR sensor and the CT were both assessed, and the closed-loop compensations resumed operation. The fused signals of the sensor were also acquired to evaluate their frequency responses. In Figure 5, the “Fusion Resp.” represents the frequency response of the fused signal. The region filled in light red in each subfigure represents the variation range of the fusion signal, with nearby annotations in red describing the percentage of variation to the passband sensitivity, *δ*. For the WODRC signal fusion sensor, *δ* = 5.88%; while, for the signal WDRC fusion sensor, *δ* = 3.76%. This result indicates that, when using sectionally distributed resistors and capacitors, the fused signal achieved greater flatness in the full measuring frequency range. For the phase variation comparison, the WODRC signal fusion sensor and WDRC had almost the same results as 4°, mainly due to the phase deviation in a low-frequency range below 5 Hz. This deviation may have been caused by a test error and could be compensated for.

Additionally, the measured frequency ranges of the TMR low-frequency sensor and the CT high-frequency sensor in Figure 5 are marked with pink and a light blue block fill, respectively. In the overlapping area within both measured frequency ranges, the block is presented as light purple. The upper 3 dB bandwidth of the TMR and *f*_TMR_ and the lower 3 dB bandwidth of the CT and *f*_CT_ are given in annotations with the same color as each measured frequency response curve. For the WODRC signal fusion sensor, *f*_TMR_ = 37.28 kHz and *f*_CT_ = 1353 Hz, while for the WDRC signal fusion sensor, *f*_TMR_ = 6893 Hz and *f*_CT_ = 305 Hz. When equipped with sectionally distributed resistors and capacitors, *f*_TMR_ and *f*_CT_ in the signal fusion sensor both dropped substantially. Nevertheless, for the DC to MHz frequency range considered in this study, the overall bandwidth of the signal fusion sensor still met the relevant requirements.

According to the results presented in Figure 5, the models of the signal fusion sensor analyzed in Section 2 fit the experimental results with standard deviations less than 2%, which confirms the validity of the aforementioned model. The deviations were mainly caused by errors in the developing and monitoring processes. Compared with the WODRC sensor, the WDRC sensor has a flatter response in the passband of the sensor.

**Figure 6:**
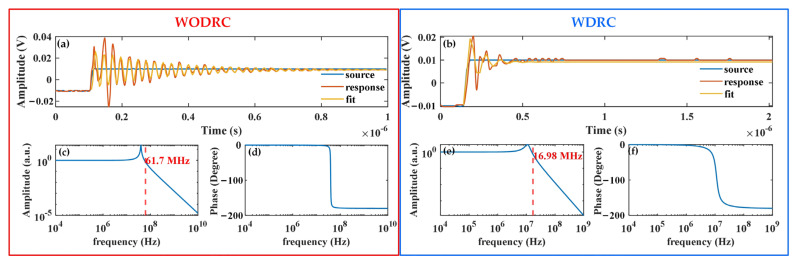
Acquiring the estimated value of the WODRC signal fusion sensor’s bandwidth (**a**,**c**,**d**) and the WDRC signal fusion sensor’s bandwidth (**b**,**e**,**f**), respectively, by fitting the data of their step responses; (**a**,**b**): the blue curves show the waveforms of the current source, the orange curves show the responses of the WODRC/WDRC signal fusion sensor to the current wave, and the yellow curves show the data fit of the responses based on the transfer function; (**c**,**e**): the amplitude frequency responses of the transfer function model with parameters acquired through data fitting in subfigures (**a**,**b**); (**d**,**f**): the phase frequency responses of the transfer function model with parameters acquired through the data fitting in subfigures (**a**,**b**).

Furthermore, Figure 5 shows that the broader frequency responses of the fused signals, including their bandwidth, cannot be evaluated. This limitation was caused by the 1 MHz bandwidth of the power amplifier in our experimental system. Consequently, another method for estimating the bandwidth was proposed. As presented in Figure 6, a step current signal with a rise time of 8 ns was injected into the primary coil of the WODRC and WDRC signal fusion sensors, and the fused signal response of each sensor was recorded using a data acquisition board. Then, a transfer function parametric model of each sensor was acquired by fitting the data of each step response and plotting the broader estimated frequency response of the corresponding transfer function. In Figure 6a,c,d present the step responses and estimated frequency responses of the WODRC sensor, while Figure 6b,e,f present the responses for the WDRC sensor. Finally, the bandwidth was estimated as 61.7 MHz for the WODRC signal fusion sensor (Figure 6c) and as 16.98 MHz for the WDRC signal fusion sensor (Figure 6e). The estimated bandwidth relationship between the fused signal of the WODRC sensor and that of the WDRC sensor was consistent with the relationship related to *f*_TMR_ and *f*_CT_ for the two sensors mentioned above. The sectionally distributed resistors and capacitors caused a decrease in the *f*_TMR_, *f*_CT_, and fused signal bandwidth of the signal fusion sensor. Nevertheless, the bandwidth of the WDRC signal fusion sensor still approached 17 MHz, far into the MHz range.

In addition, the accuracy and sensitivity of both the WODRC signal fusion sensor and WDRC sensor are evaluated by supplying a certain frequency and different amplitude of the primary current. In this process, the reference value of the current *I_r_* is read from the output voltage of the shunt divided by its nominal sensitivity, and the output voltage of the signal fusion sensors *U_o_* is simultaneously recorded. Next, we use data fitting on the linear model with the Least Mean Squares method, as follows:

(23) $$U=KI+U_{b}$$

**Figure 7:**
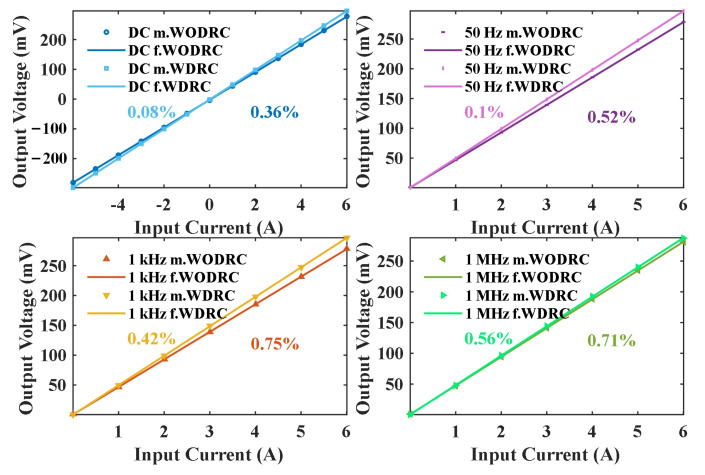
The measurement of accuracy of the WODRC/WDRC signal fusion sensor under different frequencies of the primary current (shown in the legend of each subfigure). The acquired accuracy is given in the annotation with the same color as the measured data in each subfigure: m.WODRC: measured data for the WODRC fusion sensor; f. WODRC: fit curve for the WODRC fusion sensor; m.WDRC: measured data for the WDRC fusion sensor; f. WDRC: fit curve for the WDRC fusion sensor.

In this way, the estimated value of sensitivity *K* and output deviation *U_b_* of the sensors are acquired, as given in Table 4. The output voltage *U_o_* and linear data fitting are presented in Figure 7. Then, the measured current value *I_o_* of the signal fusion sensor is obtained as follows:

(24) $$I_{o}=\left(U_{o}-U_{b}\right)/K.$$

The standard deviations in the *U_o_*, *K*, and *U_b_* are assessed via estimation to evaluate the accuracy of the signal fusion sensor. The results are listed in Table 4. In these results, each standard deviation in *K* divided by *K* itself, *σK*/*K*, is chosen to represent the accuracy of the measurement [32], which is annotated in each subfigure of Figure 7. For both types of fusion sensors, the accuracy of the signal fusion sensor was found to be lower than 1% even when the sensing frequency of the primary current was as high as 1 MHz. Overall, the accuracy of the WDRC fusion sensor was improved as high as 0.6% compared with that of the WODRC fusion sensor.

**Figure 8:**
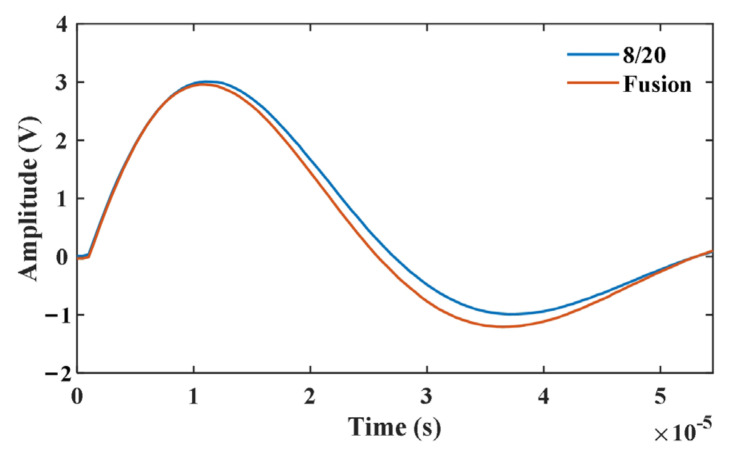
The reaction of the proposed signal WDRC fusion sensor to the 8/20 impulse current signal. The blue curve represents the standard 8/20 waveform, and the orange curve represents the acquired signal of the signal fusion sensor.

We also evaluated the proposed WDRC signal fusion sensor’s reaction to an impulse current signal using the standard 8/20 waveform. The standard 8/20 waveform was generated with a waveform generator and amplified using the wideband current amplifier to produce the impulse current signal. The reaction signal of the fusion sensor to the impulse signal was recorded along with the impulse signal itself, as presented in Figure 8. The standard deviation in the sensor reaction to the impulse signal was calculated as 0.17 V or about 5% of the maximum amplitude of the impulse signal. This evaluation verified the capability of the proposed signal fusion sensor to measure the impulse signals.

## 5. Discussions and Conclusions

In this paper, we proposed a signal fusion scheme that enables current sensors to achieve a broad and flat frequency response range from the DC to MHz range. This scheme is composed of a TMR (as the low-frequency sensor) and a CT (as the high-frequency sensor). The physical realization and measuring principles of this scheme were introduced, and the total transfer function of the entire signal fusion scheme was derived. In addition, we conducted a simulation using this scheme based on a given transfer function for verification. In the simulation results, the variation in the amplitude (phase) frequency response in the passband of the fused signal was 0.16% (1°), and the bandwidth of the fused signal extended to over 88 MHz.

Several principal prototypes were also built for testing and evaluation, including assessments of the amplitude and phase response, accuracy, sensitivity, and responses to the step and impulse signals. We also compared the performance of a WODRC signal fusion sensor with that of a WDRC fusion sensor. Equipped with sectionally distributed resistors and capacitors, the signal fusion sensor achieved reduced amplitude variation within the considered frequency range from DC to MHz, alongside improved measuring accuracy for the current at different frequencies. The bandwidth of the WODRC fusion sensor was estimated to be 61.7 MHz. The trade-off of using a WDRC fusion sensor is its narrower estimated bandwidth. However, this bandwidth still approaches 17 MHz, which is deep into the MHz range. The evaluation result shows that the signal fusion sensor achieves a flat response with an amplitude variation of less than 4% from DC to 1 MHz and an accuracy better than 0.6%, even when measuring the current at 1 MHz. The measurement of the impulse current signal using the fusion sensor achieved a 5% relative standard deviation in the impulse amplitude. In addition, calculations show that the economic cost of the proposed fusion sensor is low, making it easy to promote.

Table 5 highlights the comprehensive advantages of the signal fusion scheme proposed in this paper with a performance comparison. The current sensors made using our scheme have a wide and flat frequency response, comparatively high accuracy, and low economic cost and, as such, it may find substantial applications in the fields of technology and industrial production. The potential applications of this method include GaN third-generation semiconductor switch current measurements for power electronics, current monitoring in electric vehicles, new energy and energy storage grid connections, and grid simulation power supplies. Considering the possible real-world issues in industrial scenarios, future research on this scheme will be conducted to assess the impacts of certain phenomena such as sensor eccentricity, return conductor current, and environmental electromagnetic interference.

## Figures and Tables

**Table 2:** Coefficient of each term for the total transfer function *G*_tot_(*s*).

Coefficients	Determined by	Preferred Numerical Value
*α*	*C*, *C*_1_, *C*_2_, *C*_4_, *d*, *L*, *R*_1_, *R*_4_, *R_m_*, *τ_a_*, *τ_s_*	1.89 × 10^−40^
*β*	*C*, *C*_1_, *C*_2_, *C*_4_, *d*, *L*, *R*, *R*_1_, *R*_4_, *R_m_*, *τ_a_*, *τ_s_*	1.13 × 10^−31^
*γ*	*C*, *C*_1_, *C*_2_, *C*_4_, *d*, *L*, *R*, *R*_1_, *R*_4_, *R_m_*, *τ_a_*, *τ_s_*	4.22 × 10^−24^
*δ*	*C*, *C*_1_, *C*_2_, *C*_4_, *d*, *L*, *R*, *R*_1_, *R*_4_, *R_m_*, *τ_a_*, *τ_s_*	9.02 × 10^−19^
*ε*	*C*, *C*_1_, *C*_2_, *C*_4_, *d*, *L*, *R*, *R*_1_, *R*_4_, *R_m_*, *K_a_*, *τ_a_*, *K_s_*, *τ_s_*	2.70 × 10^−14^
*ζ*	*C*, *C*_1_, *C*_2_, *C*_4_, *d*, *L*, *R*, *R*_1_, *R*_4_, *R_m_*, *K_a_*, *τ_a_*, *K_s_*, *τ_s_*	4.81 × 10^−10^
*η*	*C*, *C*_1_, *C*_2_, *C*_4_, *d*, *R*, *R*_1_, *R*_4_, *R_m_*, *K_a_*, *τ_a_*, *K_s_*, *τ_s_*	0.0128
*θ*	*d*, *K_a_*, *K_s_*	2.51
*κ*	*C*_1_, *C*_2_, *C*_4_, *d*, *M*, *R*_1_, *R*_4_, *R_m_*, *τ_a_*, *τ_s_*	2.40 × 10^−32^
*λ*	*C*_1_, *C*_2_, *C*_4_, *d*, *M*, *R*_1_, *R*_4_, *R_m_*, *τ_a_*, *τ_s_*	9.60 × 10^−25^
*ρ*	*C*_1_, *C*_2_, *C*_4_, *d*, *M*, *R*_1_, *R*_4_, *R_m_*, *τ_a_*, *τ_s_*	1.99 × 10^−19^
*σ*	*C*, *C*_1_, *C*_2_, *C*_4_, *d*, *M*, *L*, *R*_1_, *R*_4_, *R_m_*, *K_a_*, *τ_a_*, *K_s_*, *τ_s_*	4.48 × 10^−15^
*φ*	*C*, *C*_1_, *C*_2_, *d*, *M*, *L*, *R*, *R*_1_, *R_m_*, *K_a_*, *K_s_*	1.48 × 10^−12^
*χ*	*C*, *C*_1_, *R*, *R*_1_, *R_m_*, *K_a_*, *K_s_*	0.00299
*ψ*	*R_m_*, *K_a_*, *K_s_*	0.587

**Table 3:** A basic economic cost estimation for one prototype of the proposed fusion scheme.

Component	Model	Economic Cost/USD
TMR	MultiDimension TMR 2652	2
Nanocrystalline Core	Yeke 1k107	7
Major Circuit Components	e.g., ADI amplifiers	10 in sum
Magnet Wires and Others	/	6
Total Sum		25

**Table 4:** The fit values and standard deviations for *U_o_*, *K*, and *U_b_* with different frequencies of the primary current.

	Standard Deviation	DC	50 Hz	1 kHz	1 MHz
WODRC	*σU_o_* (mV)	0.6057	0.3502	0.5092	0.484
	*K* (mV/A)	46.56	46.31	46.20	46.75
	*σK* (mV/A)	0.1680	0.2386	0.3472	0.330
	*U_b_* (mV)	−2.404	0.5264	0.4713	0.7714
	*σU_b_* (mV)	0.04491	0.06615	0.09620	0.0914
WDRC	*σU_o_* (mV)	0.1370	0.07363	0.3013	0.394
	*K* (mV/A)	49.602	49.4996	49.3584	47.91
	*σK* (mV/A)	0.03798	0.05018	0.2053	0.269
	*U_b_* (mV)	−1.380	0.1381	0.3057	0.3645
	*σU_b_* (mV)	0.01016	0.01392	0.05688	0.0745

**Table 5:** Performance comparison between the scheme proposed in this paper and other similar schemes and products.

Model or Scheme	LEM LZSR 100-TP [33]	Scheme by Chojowski et al. [19]	MultiDimension TMR7560-B [34]	This Scheme
Methods	closed-loop Hall	Hall + CT	closed-loop TMR	TMR + CT
Bandwidth	200 kHz	calculated 38 MHz	100 kHz	estimated 62 MHz/17 MHz
Accuracy	0.68%	1.87%	0.5%	0.56%
Price or Cost/USD	41	about 50	>50	25

## Data Availability

The data presented in this study are available upon reasonable request from the corresponding author.
